# Influences of Anlotinib on Cytochrome P450 Enzymes in Rats Using a Cocktail Method

**DOI:** 10.1155/2017/3619723

**Published:** 2017-12-26

**Authors:** Wei Sun, Zhe Wang, Ruimin Chen, Chengke Huang, Rui Sun, Xiaoxia Hu, Wanshu Li, Ruijie Chen

**Affiliations:** ^1^The Second Affiliated Hospital and Yuying Children's Hospital of Wenzhou Medical University, Wenzhou 325027, China; ^2^Wenzhou Medical University, Wenzhou 325035, China; ^3^Jinhua Central Hospital, Jinhua 321000, China; ^4^Ningbo Municipal Hospital of Traditional Chinese Medicine, Ningbo 315010, China

## Abstract

The present study aimed to investigate the effect of anlotinib (AL3818) on pharmacokinetics of cytochrome P450 (CYP) enzymes (CYP1A2, CYP2C6, CYP2D1, CYP2D2, and CYP3A1/2) by using five cocktail probe drugs in vivo. After pretreatment for 7 days with anlotinib (treatment group) or saline (control group) by oral administration, probe drugs phenacetin, tolbutamide, omeprazole, metoprolol, and midazolam were administered to rats by oral administration. Blood samples were obtained at a series of time-points and the concentrations of five probe drugs in plasma were determined by a UHPLC-MS/MS method. The results showed that treatment with anlotinib had no significant effect on rat CYP1A2, CYP2D2, and CYP2C6. However, anlotinib had a significant inductive effect on CYP2D1 and CYP3A1/2. Therefore, caution is needed during the concomitant use of anlotinib with other drugs metabolized by CYP2D1 and CYP3A1/2 because of potential drug-anlotinib interactions.

## 1. Introduction

Anlotinib (AL3818), a new antitumor drug, was developed by Jiangsu Chia-Tai Tianqing Pharmaceutical Co., Ltd [1, 2]. It has been approved by State Food and Drug Administration (SFDA) and China Food and Drug Administration (CFDA) and is in phase II or III clinical trial for the treatment of patients with renal cancer, non-small cell lung cancer, and gastric cancer. Receptor tyrosine kinases (RTKs) are major novel targets in anticancer molecular therapy by blocking of the tumoral angiogenesis [3, 4]. Anlotinib is a kinase inhibitor of receptor tyrosine with multitargets, especially for vascular endothelial growth factor receptor 2 (VEGFR2), VEGFR3, platelet-derived growth factor receptor *β* (PDGFR *β*), and c-Kit. It has obvious resistance to vasculogenesis and angiogenesis. The vascular endothelial growth factor (VEGF) and its receptors (VEGFRs) are crucial proteins in both physiological and pathological angiogenesis, and VEGFRs have been proved to be effective anticancer targets [5–7]. Similarly, PDGFR is an important target for novel anticancer therapeutics because it is overexpressed in a wide variety of malignancies [8].

Cytochrome P450s (CYPs) are the main catalytic enzymes for the biotransformation of a variety of exogenous chemicals, including drugs, carcinogens, and toxins, and endogenous compounds such as steroids, fatty acids, and prostaglandins [9]. CYP450 is a supergene family of drug-metabolizing enzymes responsible for the metabolism of approximately 75% of clinically administered drugs [10, 11]. The greatest contributor to the biotransformation of drugs in human is CYP3A4/5, followed by CYP2D6, CYP2C6, CYP1A2, and others [12]. It has been demonstrated that rat CYP1A2, CYP2C6, CYP2D1, CYP2D2, and CYP3A1/2 are homologous to human CYP1A2, CYP2C9, CYP2C19, CYP2D6, and CYP3A4, respectively [13, 14]. Inhibition or induction of CYPs has been recognized as one of the major causes for clinical drug-drug interactions (DDIs). Assessing DDIs, especially the evaluation of the effect of a new drug on the CYP450 enzyme activities, is essential and important to understanding inductive and inhibitory effects [15].

A previous study has mentioned that anlotinib is primarily metabolized by cytochrome P450-mediated biotransformation reactions in vitro [1]. However, the impact of anlotinib on CYP450 enzyme activities in vivo has not been reported. In the present paper, the objective of our study was to evaluate the potential effects of anlotinib on the CYP isozymes CYP1A2, CYP2C6, CYP2D1, CYP2D2, and CYP3A1/2 in rats using a cocktail approach involving five probe drugs phenacetin, tolbutamide, omeprazole, metoprolol, and midazolam. Firstly, a sensitive and specific UHPLC-MS/MS method was validated to simultaneously quantify the five substrates in rat plasma as a cocktail. Then CYP activities were assessed by comparing the pharmacokinetics of the five substrates between control and anlotinib treatment groups in vivo. We predict that the results may be useful for the clinical safety evaluation of DDIs involving anlotinib.

## 2. Materials and Methods

### 2.1. Chemicals

Phenacetin, tolbutamide, omeprazole, metoprolol, midazolam (purity > 98%), and the internal standard carbamazepine (IS) were purchased from Sigma-Aldrich Company (St. Louis, USA). Anlotinib was a gift from Jiangsu Chia-Tai Tianqing Pharmaceutical Co., Ltd. HPLC grade water was produced by a Milli-Q system from Millipore (Molsheim, France). Other chemicals (analytical grade) used in the present study were purchased from standard chemical suppliers.

### 2.2. Animals

Twelve Sprague-Dawley rats (male, 200 ± 20 g) were obtained from and housed at Laboratory Animal Research Center of Wenzhou Medical University and used to study the pharmacokinetics. Animals were housed under a natural light-dark cycle conditions with controlled temperature (22°C). All experimental procedures were approved ethically by the Animal Care and Use Committee of Wenzhou Medical University.

### 2.3. UHPLC-MS/MS Conditions

UHPLC-MS/MS with Agilent 1290 UHPLC system and 6420 series Triple-Quadrupole Tandem Mass Spectrometer (Agilent, Santa Clara, CA, USA) equipped with electrospray ionization (ESI) source operating in positive-ion mode was used to monitor the mixed compounds. Instrument control and data acquisition were performed on the MassHunter Agilent Software (version B.07.00).

Five probes and IS were separated using a ZORBAX Eclipse Plus C18 Rapid Resolution HD column (2.1 mm × 50 mm, 1.8 *μ*m) at a constant temperature of 30°C. The mobile phase consisting of A (0.1% formic acid-water, v/v) and B (acetonitrile) was ultrasonic degassed before use. The optimal gradient elution program was as follows: 0–0.3 min, 30% B; 0.3–1.3 min, linear from 30% to 50% B; 1.3–1.8 min, linear from 50% to 95% B; 1.8–2.8 min, 95% B. The posttime was 1.0 min for equilibration of the column and the total runtime was 3.8 min. The flow rate was 0.4 ml/min.

The desolvation gas (nitrogen) flow was set to 10 L/h: the capillary voltage: 4000 V; nebulizing gas and drying gas (both nitrogen): 45 psi and 350°C, respectively. The multiple reaction monitoring (MRM) mode with* m*/*z* 180.1→109.9 for phenacetin,* m*/*z* 271.1→91.0 for tolbutamide,* m*/*z* 346.1→135.9 for omeprazole,* m*/*z* 268.2→115.9 for metoprolol,* m*/*z* 326.1→290.8 for midazolam, and* m*/*z* 237.1→194.0 for IS was used for quantitative analysis.

### 2.4. Pharmacokinetics

Twelve rats were randomly divided into 2 groups (*n* = 6 per group): control group and anlotinib group. The control group was given saline by oral administration, while the anlotinib group was given anlotinib (10 mg/kg) by oral administration for 7 consecutive days. On the 8th day, 4 ml/kg of probe cocktail solution, containing phenacetin (10 mg/kg), tolbutamide (1 mg/kg), omeprazole (10 mg/kg), metoprolol (10 mg/kg), and midazolam (10 mg/kg), was given to all rats in each group.

Blood samples (0.3 ml) were obtained from the tail vein into heparinized 1.5 ml polythene tubes at 0.17, 0.5, 1, 2, 3, 4, 6, 8, 12, 24, and 48 h after oral administration of probe drugs. The samples were immediately centrifuged at 10,000*g* for 10 min. The obtained plasma samples (100 *μ*l) were stored at −80°C until analysis.

### 2.5. Statistical Analysis

Drug and statistics (DAS) software (version 3.0) was used to calculate the pharmacokinetic parameters. The main pharmacokinetic parameters of the two groups were analyzed by *t*-test using IBM SPSS statistics (version 23.0). A value of *p* < 0.05 or *p* < 0.01 was considered to be statistically significant.

## 3. Results

### 3.1. Method Validation

Calibration curves showed good linearity over the range of 10–5000 ng/mL for phenacetin (*r*^2^ = 0.9976), 20–10000 ng/mL for tolbutamide (*r*^2^ = 0.9998), 15–1500 ng/mL for omeprazole (*r*^2^ = 0.9923), 10–1000 ng/mL for metoprolol (*r*^2^ = 0.9952), and 10–1000 ng/mL for midazolam (*r*^2^ = 0.9967). The LLODs of phenacetin, tolbutamide, omeprazole, metoprolol, and midazolam were 5.43, 3.43, 5.65, 5.33, and 4.46 ng/mL, respectively.

**Figure 1:**
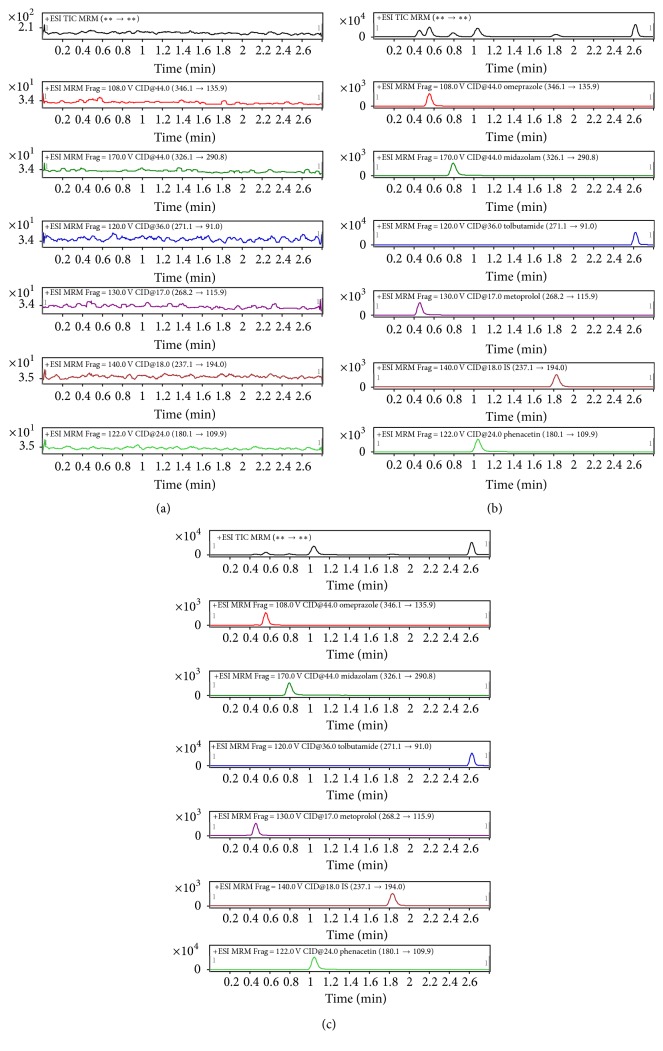
Chromatograms of five cocktail probe drugs and carbamazepine (IS) in rat plasma sample. (a) Blank plasma sample; (b) plasma sample spiked with five cocktail probe drugs and IS; (c) plasma sample obtained from rat after administration of five cocktail probe drugs.

The concentrations of phenacetin, tolbutamide, omeprazole, metoprolol, and midazolam in rat plasma were simultaneously determined by a UHPLC-MS/MS method (Figure 1). As shown in Table 1, the intraday and interday precision of the method were within 12%, and accuracy ranged from 90% to 110%. The extraction recoveries for the analytes were better than 85%. All the variations of matrix effect were in the range of 95.4–102.3%.

### 3.2. Effect of Anlotinib on Rat Hepatic CYP1A2

**Figure 2:**
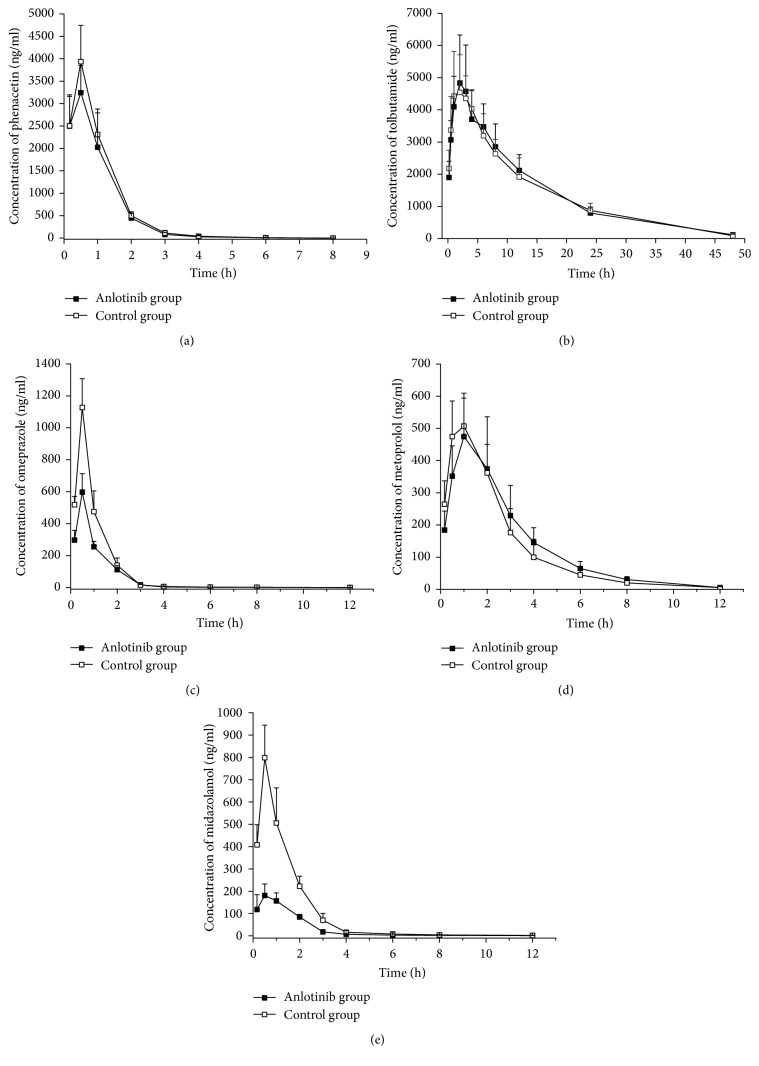
Mean plasma concentration-time curves of five probes in rats of anlotinib group and control group. (a) Phenacetin; (b) tolbutamide; (c) omeprazole; (d) metoprolol; (e) midazolam.

The main pharmacokinetic parameters of phenacetin in rats of two different groups were presented in Table 2 and Figure 2(a). There were no significant differences between the treatment group and the anlotinib group, indicating that anlotinib did not influence rat CYP1A2 activity in vivo.

### 3.3. Effect of Anlotinib on Rat Hepatic CYP2C6

Pharmacokinetic profiles of tolbutamide in control and anlotinib treatment groups were used to describe the activity of CYP2C6. As shown in Table 2 and Figure 2(b), compared with the control group, the pharmacokinetic parameters (*T*_1/2_*z*, CL*z*/*F*, MRT_(0–∞)_, AUC_(0–∞)_) of tolbutamide in the anlotinib group showed no significant change. The results indicated that anlotinib had no significantly inductive or inhibitory effect on the activity of CYP2C6 in rats.

### 3.4. Effect of Anlotinib on Rat Hepatic CYP2D1

CYP2D1 activity was evaluated by comparing pharmacokinetic behaviors of omeprazole in the study groups. The pharmacokinetic data of omeprazole in different groups were shown in Table 2 and Figure 2(c). The values of *C*_max_ and AUC_(0–∞)_ for omeprazole in the anlotinib group were 47.0% and 42.1% lower than those of control group, while CL*z*/*F* in anlotinib group was increased by 72.5%. The results indicated that metabolism of omeprazole in the treatment group was obviously accelerated, and anlotinib had the potential to induce rat hepatic CYP2D1 activity in vivo.

### 3.5. Effect of Anlotinib on Rat Hepatic CYP2D2

The activities of CYP2D2 were assessed by measuring the pharmacokinetic profiles of metoprolol in control and anlotinib treatment groups. As shown in Table 2 and Figure 2(d), compared with the control group, anlotinib did not have significant influence on *C*_max_, *T*_1/2_*z*, CL*z*/*F*, and AUC_(0–∞)_ in the two groups. Our results showed that anlotinib had no effect on the activity of CYP2D2 in rats.

### 3.6. Effect of Anlotinib on Rat Hepatic CYP3A1/2

The main parameters of anlotinib such as *T*_1/2_*z*, AUC_(0–∞)_, and CL/*F* were significantly distinct between the two groups (Table 2 and Figure 2(e)). Specifically, values of *T*_1/2_*z* and CL/*F* for midazolam in the anlotinib group were 39.5% and 227.4% higher than those of the control group, while AUC_(0–∞)_ of midazolam was diminished by 69.8% in the anlotinib group. The results showed that the metabolism of midazolam in the anlotinib treatment group was definitely accelerated, and CYP3A1/2 activity was induced by anlotinib in rats.

## 4. Discussion

In order to determine five CYP isozyme activities (CYP1A2, CYP2C6, CYP2D1, CYP2D2, and CYP3A1/2) in rat plasma, we developed a novel UHPLC-MS/MS method, consisting of phenacetin, tolbutamide, omeprazole, metoprolol, and midazolam. The determination of novel “cocktail” was validated in terms of linearity, accuracy, precision, and recovery and appropriate for studying a considerable number of samples. The method was successfully applied in the pharmacokinetic study.

Anlotinib is a new antitumor drug in phase II or III clinical trial, which works by inhibiting the tyrosine kinases. There are many receptor tyrosine kinases already on the market, such as imatinib, erlotinib, gefitinib, and sunitinib [16–18]. Imatinib is a cytochrome of CYP3A4 and CYP2C8 substrate, which can markedly increase plasma concentrations of the CYP3A4 substrate and reduce hepatic CYP3A4 activity in humans [19]. Previous in vitro study [20] showed that erlotinib was metabolized mainly by CYP3A4, with a subordinate contribution from CYP1A2. However, the metabolism of anlotinib was not completely consistent with imatinib and erlotinib.

The pharmacokinetic results indicated *C*_max_, *T*_1/2_*z*, CL*z*/*F*, AUC_(0–∞)_, and CL/*F* of three probes phenacetin, tolbutamide, and metoprolol were not decreased or increased during the concomitant use of anlotinib in the rats. Therefore, there were no statistical differences in pharmacokinetics of phenacetin, tolbutamide, and metoprolol between two groups, and the results showed that the CYP1A2, CYP2C6, and CYP2D2 activities were not significantly affected by anlotinib. The present study also focused on the effect of anlotinib on the activities of CYP2D1 and CYP3A1/2, and the main pharmacokinetics of omeprazole and midazolam were significantly changed. According to our results, investigations of CYP2D1 and CYP3A1/2 activities also showed that CYP2D1 and CYP3A1/2 were induced by anlotinib.

The purpose of the study was to evaluate the potential influences of anlotinib on the enzyme activities of CYPs in rats (CYP1A2, CYP2C6, CYP2D1, CYP2D2, and CYP3A1/2). Five probe drugs phenacetin, tolbutamide, omeprazole, metoprolol, and midazolam were selected as specific substrates for rat CYP1A2, CYP2C6, CYP2D1, CYP2D2, and CYP3A1/2, respectively. Also, phenacetin, tolbutamide, omeprazole, metoprolol, and midazolam were demonstrated to be selective substrates of human CYP1A2, CYP2C9, CYP2C19, CYP2D6, and CYP3A4. Hence, the effects of anlotinib determined in rat could be speculated to human in clinical use.

## 5. Conclusions

Anlotinib had a significant inductive effect on CYP2D1 and CYP3A1/2. Therefore, caution is needed during the concomitant use of anlotinib with other drugs metabolized by CYP2D1 and CYP3A1/2 because of potential drug-anlotinib interactions.

## Figures and Tables

**Table 1:** The interday and intraday precision, accuracy, and recovery for five probes.

Compound	Nominal concentration (ng/mL)	Interday precision measured (ng/mL)	RSD (%)	Accuracy RE (%)	Intraday precision measured (ng/mL)	RSD (%)	Accuracy RE (%)	Recovery mean ± SD (%)	RSD (%)
Phenacetin	10	9.9 ± 0.9	9.4	−1.0	9.5 ± 0.5	5.4	−4.6	90.5 ± 2.8	3.1
	250	237.0 ± 16.1	6.8	−5.2	240.2 ± 17.0	7.1	−3.9	89.8 ± 5.0	5.6
	5000	5055.4 ± 355.6	7.0	1.1	5151.4 ± 66.0	1.3	3.0	90.5 ± 4.1	4.5

Tolbutamide	20	21.0 ± 1.6	7.7	5.1	20.5 ± 0.9	4.2	2.5	88.7 ± 2.1	2.3
	500	504.4 ± 26.6	5.3	0.9	493.0 ± 15.7	3.2	−1.4	87.2 ± 5.2	6.0
	10000	9793.4 ± 632.2	6.5	−2.1	9653.2 ± 383.8	4.0	−3.5	91.7 ± 6.0	6.5

Omeprazole	15	15.3 ± 0.6	3.7	1.9	15.5 ± 0.6	3.8	3.6	86.1 ± 3.7	4.3
	150	147.8 ± 10.8	7.3	−1.7	147.6 ± 13.6	9.2	−1.6	87.8 ± 5.6	6.4
	1500	1570.0 ± 71.7	4.6	4.7	1570.0 ± 48.2	3.1	4.7	91.0 ± 2.4	2.7

Metoprolol	10	9.7 ± 0.4	4.4	−2.8	9.7 ± 0.5	4.7	−2.8	85.3 ± 2.5	2.9
	100	100.2 ± 7.4	7.4	0.2	100.6 ± 6.1	6.1	0.6	91.4 ± 7.5	8.2
	1000	1064.0 ± 94.5	8.9	6.4	1058.6 ± 95.9	9.1	5.9	88.4 ± 6.5	7.3

Midazolam	10	9.5 ± 0.3	2.7	−5.0	9.9 ± 0.6	5.8	−1.4	89.5 ± 5.7	6.3
	100	102.4 ± 8.6	8.5	2.4	109.0 ± 8.6	7.9	9.0	86.3 ± 4.8	5.6
	1000	1022.0 ± 116.5	11.4	2.2	1017.8 ± 46.8	4.6	1.8	88.8 ± 6.9	7.7

**Table 2:** Main pharmacokinetic parameters of five in rats (*n* = 6, mean ± SD).

Probe drugs	Parameters	*T*_1/2_ *z*, h	CL*z*/*F*, L/h/kg	*C*_max_, ng/ml	MRT_(0–*∞*)_, h	AUC_(0–*t*)_, ng/ml·h	AUC_(0–*∞*)_, ng/ml·h
Phenacetin	Control	0.81 ± 0.18	2.19 ± 0.40	3936.43 ± 806.97	0.93 ± 0.08	4689.91 ± 860.47	4691.26 ± 861.16
	Anlotinib	1.05 ± 0.32	2.57 ± 0.69	3246.16 ± 601.48	0.92 ± 0.08	4088.86 ± 928.94	4092.10 ± 930.31
Tolbutamide	Control	8.14 ± 1.16	0.15 ± 0.03	5313.03 ± 947.57	12.24 ± 1.20	66632.93 ± 10801.35	67830.74 ± 11352.40
	Anlotinib	8.41 ± 1.62	0.15 ± 0.03	4919.48 ± 1517.46	12.38 ± 0.95	67955.32 ± 13445.81	69497.87 ± 14184.04
Omeprazole	Control	2.09 ± 0.44	8.99 ± 1.42	1126.62 ± 181.00	0.96 ± 0.08	1132.06 ± 163.11	1133.42 ± 163.50
	Anlotinib	2.42 ± 0.40	15.51 ± 2.18^*∗∗*^	597.13 ± 115.68^*∗∗*^	1.06 ± 0.06^*∗∗*^	655.07 ± 99.34^*∗∗*^	656.25 ± 99.82^*∗∗*^
Metoprolol	Control	1.87 ± 0.24	6.98 ± 1.59	531.67 ± 66.28	2.39 ± 0.38	1494.16 ± 385.75	1508.49 ± 395.46
	Anlotinib	1.69 ± 0.20	6.86 ± 2.69	494.31 ± 118.60	2.81 ± 0.16	1602.56 ± 504.44	1616.59 ± 509.09
Midazolam	Control	2.15 ± 0.47	8.87 ± 1.63	798.41 ± 145.91	1.38 ± 0.15	1163.23 ± 263.09	1167.74 ± 267.42
	Anlotinib	3.00 ± 0.49^*∗*^	29.04 ± 4.58^*∗∗*^	191.59 ± 41.80^*∗∗*^	1.69 ± 0.21^*∗*^	348.48 ± 59.32^*∗∗*^	352.19 ± 60.05^*∗∗*^

^*∗*^Significantly different from control, *p* < 0.05. ^*∗∗*^Significantly different from control, *p* < 0.01.
